# Improving ultrasound-based prostate volume estimation

**DOI:** 10.1186/s12894-019-0492-2

**Published:** 2019-07-24

**Authors:** Saro Aprikian, Murilo Luz, Fadi Brimo, Eleonora Scarlata, Lucie Hamel, Fabio L. Cury, Simon Tanguay, Armen G. Aprikian, Wassim Kassouf, Simone Chevalier

**Affiliations:** 1 Urologic Oncology Research Group, Cancer Research Program, Research Institute (RI) of McGill University Health Center (MUHC), Glen Campus, E M2.2210, 1001 Blvd Décarie, Montréal, Qc H4A 3J1 Canada; 2 Department of Surgery (Urology Division), MUHC and McGill University, Montreal, Canada; 3 Department of Pathology, MUHC and McGill University, Montreal, Canada; 4 Department of Radiation Oncology, MUHC and McGill University, Montreal, Canada; 5 Department of Oncology, MUHC and McGill University, Montreal, Canada; 6 Department of Medicine, MUHC and McGill University, Montreal, Canada

**Keywords:** Prostate volume, Trans-rectal ultrasound, Ellipsoid, Bullet, Formula, Coefficient

## Abstract

**Background:**

To define a new coefficient to be used in the formula (Volume = L x H x W x Coefficient) that better estimates prostate volume using dimensions of fresh prostates from patients who had transrectal ultrasound (TRUS) imaging prior to prostatectomy.

**Methods:**

The prostate was obtained from 153 patients, weighed and measured to obtain length (L), height (H), and width (W). The density was determined by water displacement to calculate volume. TRUS data were retrieved from patient charts. Linear regression analyses were performed to compare various prostate volume formulas, including the commonly used ellipsoid formula and newly introduced bullet-shaped formula.

**Results:**

By relating measured prostate volumes from fresh prostates to TRUS-estimated prostate volumes, 0.66 was the best fitting coefficient in the (L x H x W x Coefficient) equation. This newfound coefficient combined with outlier removal yielded a linear equation with an R^2^ of 0.64, compared to 0.55 and 0.60, for the ellipsoid and bullet, respectively. By comparing each of the measured vs. estimated dimensions, we observed that the mean prostate height and length were overestimated by 11.1 and 10.8% using ultrasound (*p* < 0.05), respectively, while the mean width was similar (*p* > 0.05). Overall, the ellipsoid formula underestimates prostate volumes by 18%, compared to an overestimation of 4.6 and 5.7% for the bullet formula and the formula using our coefficient, respectively.

**Conclusions:**

This study defines, for the first time, a coefficient based on freshly resected prostates as a reference to estimate volumes by imaging. Our findings support a bullet rather than an ellipsoid prostate shape. Moreover, substituting the coefficient commonly used in the ellipsoid formula by our calculated coefficient in the equation estimating prostate volume by TRUS, provides a more accurate value of the true prostate volume.

## Background

Prostate volume is an important parameter used by clinicians to better manage patients with lower urinary tract symptoms (LUTS) or cancer. The increase in prostate volume with age is correlated with the development of benign prostatic hyperplasia (BPH), which can potentially obstruct the bladder outlet, and necessitate interventions [1]. Knowing the volume of the prostate can help surgeons decide on pharmacological treatments or possibly optimal surgical modalities to resect sufficient prostatic tissue to relieve or improve LUTS. Moreover, the prostate volume is of utility in prostate cancer detection and management as it can be used in conjunction with serum prostatic specific antigen (PSA) to define the PSA density, a clinically relevant parameter for decision-making [2]. In addition, the measurement of prostatic volume is also required in other fields such as radiation oncology, as patients might not be deemed candidates for brachytherapy or stereotactic body radiotherapy (SBRT) if prostate volumes are > 60 cc and > 80 cc, respectively. Hence, estimating the actual prostate volume with accuracy is required for proper medical and surgical management of prostatic diseases as well as for ablative procedures.

Ultrasonography, and more specifically transrectal ultrasonography (TRUS), is the most commonly used tool to estimate prostate volume [3]. The ultrasonograph computes the total volume by measuring the length (L), height (H) and width (W) of the gland and multiplying the product by a coefficient of *π*/6 (0.52), also known as the prolate ellipsoid formula [4]. The same formula can also be used in Magnetic Resonance Imaging (MRI) modalities for the estimation of prostate volume [4]. Another geometrical model known as the bullet formula (L x H x W × 5*π*/24) or (L x H x W × 0.65) was proposed in 2009 as a potentially superior formula in estimating prostatic volume [5]. Interestingly, existing studies examining the accuracy of these methods in measuring true prostate volume have reported conflicting evidence as to which formula is more accurate. For instance, Lee et al. had concluded that the ellipsoid formula is adequate and accurate enough in estimating prostatic volume using either TRUS or MRI [4]. However, Rodriguez et al. rather concluded that the ellipsoid formula consistently underestimates prostate size by more than 10% at least 80% of the time [6]. Furthermore, and in support of the latter findings, MacMahon et al. have demonstrated that the ellipsoid formula, on average, underestimated prostate volume by 17% [5].

These conflicting studies, along with the lack of comparison to true prostate volumes measured from fresh prostate-based studies, prompted the present investigation aiming to determine the best possible coefficient to be used in imaging-based volume estimation obtained from the dimensions of fresh prostates.

## Methods

### Patient cohort

Radical prostatectomy was performed at the McGill University Health Center on 153 patients with localized prostate cancer between the ages of 44 to 76. All had undergone standardized TRUS examination of the prostate by experienced ultrasonographers prior to surgery. Patients had signed consent forms approved by the McGill Ethics Committee for the use of their data for research.

### Prostate measurements

Within 10 min of surgical removal, each prostate was trimmed to remove seminal vesicles and surrounding fat tissues, weighed, and measured by two examiners with a 15 cm ruler using standard operating procedures in Pathology to obtain the prostate dimensions, L (apex to base), H (anterior to posterior) and W (lateral to lateral). The prostate was subsequently inked and further processed fresh for banking and formaldehyde-fixed for pathological examination. All parameters were measured prospectively by the same two individuals over time.

A series of 8 fresh prostates were used in water displacement experiments, according to Archimedes’ Principle, to determine the mean density. With this newfound density, prostate weights were directly converted to volumes and considered as *measured* volumes.

TRUS measurement data were retrieved from patient medical files. They comprised the individual L, H, and W dimensions as well as the *estimated* volume determined using the standard prolate ellipsoid formula. The true prostate dimensions were used in relation with the true measured volumes post-surgery in order to determine a new coefficient that best relates the two volumes. The measured dimensions by TRUS were then incorporated in all three different formulas (ellipsoid, bullet and our newfound formula) to compare volumes.

### Statistical analyses

Multiple linear regression analyses, Bland-Altman and box plots as well as Student t-tests were performed to compare prostate measured volumes with estimated volumes in addition to comparing the individual dimensional measurements from TRUS with the ones measured after surgical resection of the prostate. Differences were considered significant at *p* ≤ 0.05.

## Results

### Basic characteristics of freshly resected prostates

The mean prostate weight was 47.1 g, ranging from 22.2 g to 115.9 g for the 153-patient cohort. The weight range of the series of 8 prostates used in the water displacement experiments, that are representative of radical prostatectomy cohorts, was from 21.0 g to 82.2 g, with a mean weight of 50.2 g and diverging from the overall cohort by 6.6%. Based on the new ISUP Grade Grouping (GG) [7] the 8 prostates were classified as: 2 GG 1; 5 GG 2; and 1 GG 3; pT2c to pT3b; 5 to 10% tumor volume. Using Archimedes’ Principle, we determined the density of the average prostate to be 1.02 ± 0.01 g/cc.

### Relationship between measured and estimated prostate volumes

The estimated volume from TRUS imaging assumes an ellipsoid geometrical shape of the prostate using the formula (V = L x H x W x Coefficient). In order to identify the best coefficient for this series of 153 fresh prostate specimens, we used the measured prostate weight converted to measured prostate volume by using 1.02 g/cc as the density, as defined above. Thus, the mean measured volume of our cohort was 48.1 cc (range 22.6-118.3 cc).

Measured prostate volumes along with TRUS-obtained prostate dimensions were then used to calculate a new coefficient from the rearranged algebraic formula: Coefficient = V/L x H x W, where L, H and W were all obtained by TRUS, and V is the measured prostatic volume of fresh prostates, obtained just after surgery, as mentioned above. This calculation was performed for each of the 153 prostates, which led to a calculated mean coefficient of 0.66.

**Fig. 1:**
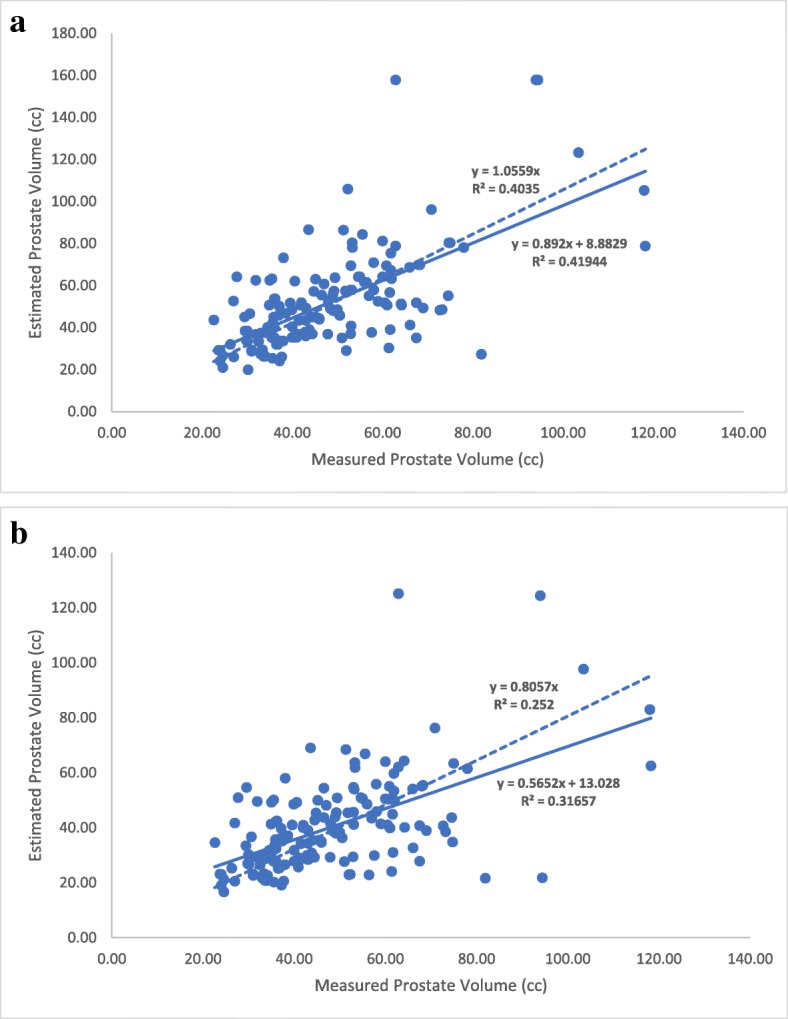
Linear regression analyses of estimated vs. measured prostate volumes using (**a**) the new formula with 0.66 as a coefficient and (**b**) the ellipsoid formula. Solid line represents the linear regression with a constant term. Dashed line represents the linear regression passing through the intercept

Linear regression plots were created in order to compare the newfound coefficient of 0.66 with the ellipsoid coefficient of 0.52. Figure 1a (solid line) shows that plotting estimated prostate volumes against measured prostate volumes using 0.66 as a coefficient yielded an equation of y = 0.892x + 8.8829 with an R^2^ value of 0.42 (*p*-value = 0.043; lower 95% confidence interval/CI = 0.27 and upper 95% CI = 17.49). By performing the same analysis using 0.52 as the coefficient, the equation generated was y = 0.5652x + 13.028 with R^2^ = 0.32 (Fig. 1b).

**Fig. 2:**
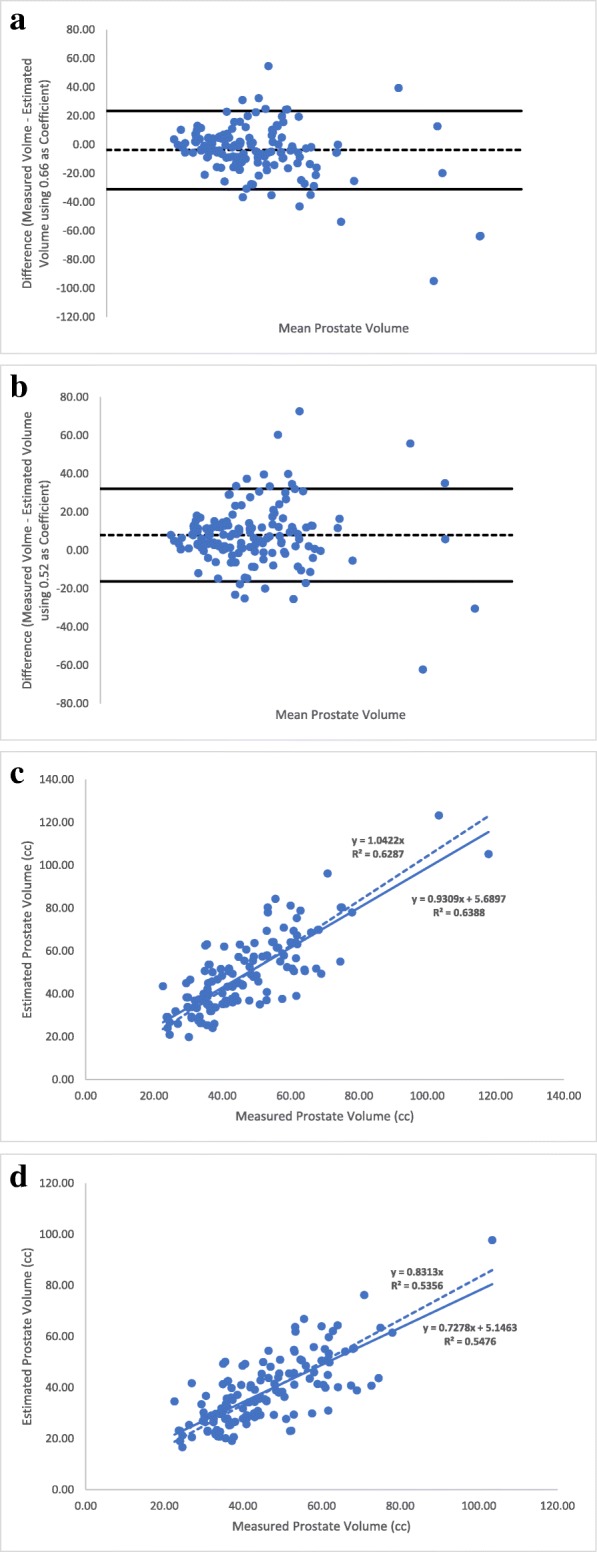
Bland-Altman plots for outlier removal when using (**a**) the new formula with 0.66 as a coefficient and (**b**) the ellipsoid formula. Linear regression analyses of estimated vs. measured prostate volumes using (**c**) the new formula with 0.66 as a coefficient, after outlier removal and (**d**) the ellipsoid formula, after outlier removal. In (**c**) and (**d**), solid line represents the linear regression with a constant term. Dashed line represents the linear regression passing through the intercept

While values were relatively well fitting in the regression, outliers were observed. Bland-Altman analyses (Fig. 2a and b) were used to keep values within 1.5 standard deviations. This type of analysis has as purpose to assess the agreement between two quantitative methods of measurement [8]. This resulted in 18 out of 153 (11.7%) outliers, which were consequently removed. The new mean measured prostate volume of the updated cohort of 135 cases was 46.2 cc (range 22.6–118.0 cc).

New regression analyses were performed with the reduced cohort, using 0.66 and 0.52 as coefficients. Figure 2c shows that the updated equation with 0.66 as the coefficient was improved to y = 0.9309x + 5.6897 with an R^2^ of 0.64, compared to the analysis using the ellipsoid coefficient, which yielded an equation of 0.7278x + 5.1463 and an R^2^ of 0.55 (Fig. 2d), thereby demonstrating the superiority of 0.66 as the coefficient to better estimate prostate volumes using TRUS dimensions.

**Fig. 3:**
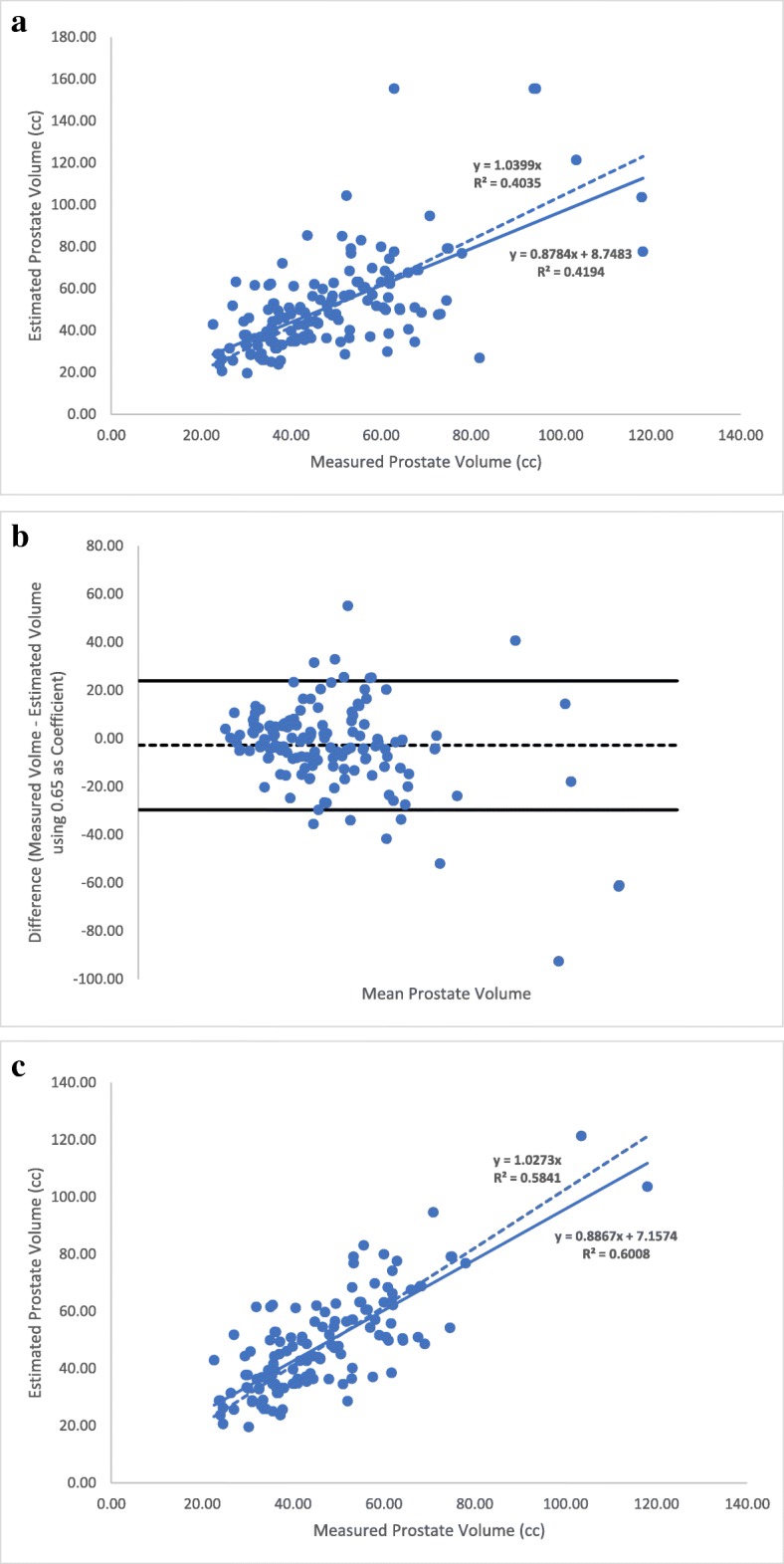
Linear regression analysis of estimated vs. measured prostate volumes using the bullet formula (**a**) with the full cohort. **b** Bland-Altman plot for outlier removal when using the bullet formula. **c** Linear regression analysis of estimated vs. measured prostate volumes using the bullet formula with the updated cohort, after outlier removal. In (**a**) and (**c**), solid line represents the linear regression with a constant term. Dashed line represents the linear regression passing through the intercept

The second step was to assess the accuracy of our coefficient compared to the bullet formula, L x H x W × 5*π*/24, as proposed by MacMahon et al. [5]. In Fig. 3a, we repeated the same type of regression analyses using the bullet formula for the entire cohort (*n* = 153; as in Fig. 1a). The equation generated was y = 0.8784x + 8.7483 with an R^2^ value of 0.42. The Bland-Altman analysis was also performed (Fig. 3b) as above, allowing removal of outliers, reducing the cohort to 138 prostates. The new regression analysis yielded an equation of y = 0.8867x + 7.1574 with an R^2^ value of 0.60 (Fig. 3c), a value closer to the one obtained in Fig. 2c using 0.66 as a coefficient, but with a lower slope value 0.8784 for the bullet coefficient 0.65 vs. 0.9309 for the new coefficient 0.66.

Altogether these findings suggest that our prostate-derived coefficient of 0.66 is more accurate than that of the ellipsoid formula (0.52) and slightly better than the coefficient of the bullet formula (0.65) in estimating true prostate volume.

### Comparison of measured and TRUS-estimated prostate dimensions

**Fig. 4:**
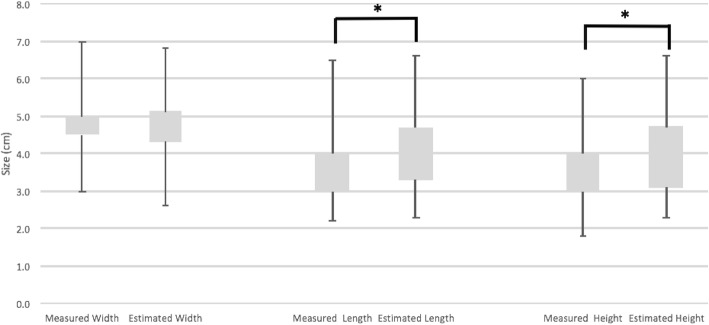
Box plot of individual prostate dimensions, estimated vs. measured. * *p* ≤ 0.05

The next step was to examine differences in individual prostate dimensions according to the method used to obtain them. We compared each dimension (L, H, W) estimated by ultrasound prior to surgery vs. the same respective prostate dimensions measured post-surgery from our initial cohort of 153 patients. This comparison is shown in Fig. 4. We observed that the mean length of the prostate was overestimated by 10.8% using ultrasound (4.1 cm vs. 3.7 cm) (*p* < 0.05). The mean height was overestimated using TRUS by 11.1% (4.0 cm vs. 3.6 cm) (*p* < 0.05). Finally, the width of the gland estimated by TRUS was fairly similar to the measured value (4.7 cm vs. 4.8 cm) (*p* > 0.05).

### Comparison of measured prostate volume and TRUS – estimated prostate volume

We further examined the percent-difference in volumes between measured and estimated volumes using the three formulas for our primary cohort (*n* = 153). In comparison to the measured volume, the ellipsoid formula revealed an underestimation of the volume by a mean of 8.80 cc (18%), whereas the bullet formula overestimated prostate volumes by 1.97 cc (4.6%). Finally, when comparing measured prostate volumes to TRUS-estimated volumes using our new formula with 0.66 as the coefficient, there was an overestimation by a mean of 2.76 cc (5.7%).

Prostates were next grouped by size to compare the performance of the three coefficients in estimating prostate volumes. For the 37 larger prostates with measured volumes greater than 60 cc (mean = 76.0 cc), the volume differences between measured vs. TRUS-calculated by ellipsoid, bullet, or with 0.66 as the coefficient were − 22.74 cc (− 30%), − 6.07 cc (− 8%), and − 5.00 cc (− 6.6%), respectively. Performing the same analysis for the 13 smaller prostates (< 30 cc) (mean = 26.7 cc), differences were 3.36 cc (12.6%) for the ellipsoid, 8.6 cc (32.2%) for the bullet, and 9.14 cc (34.2%) using 0.66 as the coefficient. For the medium sized prostates (between 30 and 60 cc) (mean = 42.7 cc), differences for ellipsoid, bullet, and with 0.66 as the coefficient were 5.78 cc (14%), − 3.57 cc (− 8.3%) and − 4.31 cc (− 10%), respectively. These results suggest that the ellipsoid formula is more conformant to smaller prostates below 30 cc, while the bullet and our new formula with 0.66 as the coefficient conform better to medium- to larger-sized prostates of over 30 cc and as observed in most patients consulting for BPH and prostate cancer.

## Discussion

The estimation of prostate volume is valuable for clinicians seeing patients with potential prostatic diseases, helping them in both diagnosis and treatment. As such, TRUS remains the main method applied routinely in the clinic to assess prostate volumes. Thus far, geometrical models were used to describe prostate shape, assuming the gland to be a prolate ellipsoid and more recently, a bullet. The present study is the first to prospectively use fresh prostate specimens as a reference to define a new coefficient of 0.66, which performed better than the universally adopted coefficient of 0.52 from the ellipsoid formula to estimate prostate volumes. Moreover, the new coefficient predicts a model closer to a bullet-shaped prostate based on 0.65 as its coefficient. Therefore, we suggest using 0.66 as the coefficient in the eq. (V = L x H x W x Coefficient) for image-based prostate volume estimation.

Among reasons in support of this proposition is that the coefficient derived from freshly obtained and trimmed prostates in our study is quite representative of patients consulting clinicians in general. For instance, this cohort was part of a larger cohort of 604 consecutive patients with a similar age distribution (mean = 61.3 years old, range = 44–76 years old) undergoing radical prostatectomy for localized prostate cancer at our centre. Unfortunately, TRUS measurement data were not available for most patients who were often referred for surgery from other centres. Nevertheless, all prostates were similarly processed, and their mean weight was similar: 45.1 g (range = 17.1 g–165.6 g). Among additional factors supporting the adoption of the prostate-derived 0.66 coefficient is the fresh status of specimens, rapidly processed and measured prospectively after excision, which is close to the in vivo situation. Often times in other studies, measurements are done after formaldehyde fixation, a process known to affect the prostate weight and volume [9].

The comparison of the 0.66 coefficient with the ellipsoid and bullet coefficients showed superiority of the prostate-derived coefficient, performing better than the universally-adopted 0.52 value and being closer to the bullet formula using 0.65 as the coefficient. Indeed, the equation obtained between measured and estimated prostate volumes always yielded a higher slope and a better correlation with the 0.66 coefficient over the two others, before and after the removal of outliers. Our results are somewhat similar to a study performed on 73 prostate samples using the ellipsoid formula, showing an almost identical slope value, 0.933 vs. 0.931 in the present study, but differing by the previously reported higher R^2^ value of 0.847 [4]. This discrepancy may be related to cohort size which was significantly larger in our study (*n* = 153 vs. 73), and was composed of a greater number of prostates with larger volumes, 48.1 cc in our study vs. 39.2 cc in the study done by Lee et al. As mentioned above, the average volume of the prostate in patients undergoing radical prostatectomy for localized prostate cancer at our center is approximately 50.0 cc.

Our study showed that measurements done by experienced ultrasonographers using TRUS as compared to our pathologists’ measurements by ruler on the same prostate in the fresh setting, underestimated height (4.0 cm vs. 3.6 cm) as well as length (4.1 cm vs. 3.7 cm), while the width was similar in both cases (4.7 cm vs. 4.8 cm). These findings are slightly different than that of an earlier report carried out on 87 prostates, which indicated an underestimation of height using TRUS vs. pathological dimensions (3.2 cm vs. 3.7 cm), and width (4.8 cm vs. 5.2 cm) but no difference in length [6].

Our study also demonstrated that the ellipsoid formula underestimates prostate volume by a mean of 18% when using TRUS imaging. The extent of underestimation of TRUS-measured prostate volume using the ellipsoid formula has been reported in a prior study to be greater than 10% and shown to underestimate true prostate volumes 80% of the time [6]. In comparison, analyses with our coefficient in the formula (V = H x L x W × 0.66) provides a more accurate estimate of prostate volumes with an overestimation of 5.7%. In addition, a larger study comparing TRUS estimation of prostate volumes using the ellipsoid formula with 318 prostatectomy specimens showed a 9.1% underestimation for prostates with a mean volume of 37.3 cc [10]. Once more, the prostates in the latter study were smaller than in our series, 11 cc less than the average 48.1 cc prostates we studied. Also, it is important to emphasize that in that study the measurements of the prostatectomy specimens were retrieved retrospectively from pathology reports as opposed to the prospective nature in our study focused on true prostate volume determination. Furthermore, it is unclear whether seminal vesicles were removed, measurements were done on fresh vs. fixed specimens, or if there were one or several health care professionals measuring prostate dimensions and recording data. A main limitation in all studies, including our own, is that TRUS data for prostate dimensions and volumes were retrieved from patient medical charts since it was part of routine tests done in the clinic and not part of a prospective research project. Finally, Scott et al. evaluated the reliability of total prostate and transition zone volume measurements by TRUS among three examiners with various levels of experience and, as expected, found that more experienced examiners had better reproducibility [11].

Our new coefficient estimates a model closer to a bullet-shaped prostate, with its formula developed via ultrasound planimetry creating 3D images for brachytherapy [5]. The comparison of prostate volumes using the 5*π*/24 coefficient (0.65) of the bullet formula with our coefficient of 0.66 supports a slightly higher accuracy with our prostate-derived 0.66 coefficient, with a better slope value (0.931 vs. bullet slope of 0.887) and correlation coefficient (R^2^ = 0.64 vs. 0.60 for the bullet). The bullet formula was reported to yield a R^2^ of 0.935 when comparing planimetry-based volumes but for smaller prostates (mean of 29.4 cc) in a smaller sample set (60 cases) [5]. More importantly, our study is the first to validate the bullet formula with prospectively measured fresh prostate specimens.

By comparing all formulas in three different prostate volume subgroups: small (< 30 cc), medium (> 30 cc < 60 cc) and large (> 60 cc), we observed that the ellipsoid formula estimates measured volume better when studying smaller prostates, while the bullet formula and our prostate-derived formula using 0.66 as the coefficient better estimates true volume in the case of medium to large prostates, as observed in the majority of men consulting for BPH or prostate cancer. Nevertheless, an external validation study should be conducted in order to confirm our findings before it can be used clinically.

Although we believe that our new coefficient is better suited for clinical use in men consulting for BPH and prostate cancer, we appreciate and understand the challenges in changing the standard practice of the universally accepted, geometrically defined ellipsoid formula and its coefficient of 0.52. This is highlighted by the fact that the bullet formula and its superior coefficient of 0.65, as established in 2009, and which has been shown to be superior to the ellipsoid formula has not been adopted in clinical practice. The adoption of our coefficient, the first to be prostate-derived, will depend on the diffusion of the present findings in the medical community and its testing and applicability by specialists advocating for the integration of our coefficient in medical devices or equipments designed for prostate volume estimation.

## Conclusions

Our prospective study on freshly obtained prostates as the reference defines a coefficient of 0.66 to be superior in estimating prostate volume in the eq. V = L x H x W x Coefficient when using TRUS. This new coefficient not only provides a more accurate estimation of true prostate volumes than the universally established ellipsoid coefficient, but also supports the suggestion that the prostate shape is closer to that of a bullet.

## Data Availability

The datasets analyzed during the current study are available from the corresponding author on reasonable request.

## Abbreviations

BPH: Benign prostatic hyperplasia
CI: Confidence interval
GG: ISUP Grade Grouping
H: Height
L: Length
LUTS: Lower urinary tract symptoms
MRI: Magnetic Resonance Imaging
PSA: Prostatic specific antigen
SBRT: Stereotactic body radiotherapy
TRUS: Transrectal Ultrasound
W: Width

## References

[1] Nickel JC (2003). Benign prostatic hyperplasia: does prostate size matter?. Rev Urol.

[2] Jue JS, Barboza MP, Prakash NS, Venkatramani V, Sinha VR, Pavan N, Nahar B, Kanabur P, Ahdoot M, Dong Y (2017). Re-examining prostate-specific antigen (PSA) density: defining the optimal PSA range and patients for using PSA density to predict prostate Cancer using extended template biopsy. Urology.

[3] Harvey CJ, Pilcher J, Richenberg J, Patel U, Frauscher F (2012). Applications of transrectal ultrasound in prostate cancer. Br J Radiol.

[4] Lee JS, Chung BH (2007). Transrectal ultrasound versus magnetic resonance imaging in the estimation of prostate volume as compared with radical prostatectomy specimens. Urol Int.

[5] MacMahon PJ, Kennedy AM, Murphy DT, Maher M, McNicholas MM (2009). Modified prostate volume algorithm improves transrectal US volume estimation in men presenting for prostate brachytherapy. Radiology.

[6] Rodriguez E, Skarecky D, Narula N, Ahlering TE (2008). Prostate volume estimation using the ellipsoid formula consistently underestimates actual gland size. J Urol.

[7] Epstein JI, Egevad L, Amin MB, Delahunt B, Srigley JR, Humphrey PA (2016). The 2014 International Society of Urological Pathology (ISUP) consensus conference on Gleason grading of prostatic carcinoma: definition of grading patterns and proposal for a new grading system. Am J Surg Pathol.

[8] Giavarina D (2015). Understanding bland Altman analysis. Biochem Med (Zagreb).

[9] Schned AR, Wheeler KJ, Hodorowski CA, Heaney JA, Ernstoff MS, Amdur RJ, Harris RD (1996). Tissue-shrinkage correction factor in the calculation of prostate cancer volume. Am J Surg Pathol.

[10] Paterson NR, Lavallee LT, Nguyen LN, Witiuk K, Ross J, Mallick R, Shabana W, MacDonald B, Scheida N, Fergusson D (2016). Prostate volume estimations using magnetic resonance imaging and transrectal ultrasound compared to radical prostatectomy specimens. Can Urol Assoc J.

[11] Sech S, Montoya J, Girman CJ, Rhodes T, Roehrborn CG (2001). Interexaminer reliability of transrectal ultrasound for estimating prostate volume. J Urol.

